# A Type 1 Diabetes Polygenic Score Is Not Associated With Prevalent Type 2 Diabetes in Large Population Studies

**DOI:** 10.1210/jendso/bvad123

**Published:** 2023-10-05

**Authors:** Shylaja Srinivasan, Peitao Wu, Josep M Mercader, Miriam S Udler, Bianca C Porneala, Traci M Bartz, James S Floyd, Colleen Sitlani, Xiquing Guo, Jeffrey Haessler, Charles Kooperberg, Jun Liu, Shahzad Ahmad, Cornelia van Duijn, Ching-Ti Liu, Mark O Goodarzi, Jose C Florez, James B Meigs, Jerome I Rotter, Stephen S Rich, Josée Dupuis, Aaron Leong

**Affiliations:** Division of Pediatric Endocrinology, University of California at San Francisco, San Francisco, CA 94158, USA; Department of Biostatistics, Boston University School of Public Health, Boston, MA 02215, USA; Department of Medicine, Harvard Medical School, Boston, MA 02115, USA; Programs in Metabolism and Medical & Population Genetics, Broad Institute of Harvard & Massachusetts Institute of Technology, Cambridge, MA 02142, USA; Center for Genomic Medicine and Diabetes Unit, Massachusetts General Hospital, Boston, MA 02114, USA; Department of Medicine, Harvard Medical School, Boston, MA 02115, USA; Programs in Metabolism and Medical & Population Genetics, Broad Institute of Harvard & Massachusetts Institute of Technology, Cambridge, MA 02142, USA; Center for Genomic Medicine and Diabetes Unit, Massachusetts General Hospital, Boston, MA 02114, USA; Division of General Internal Medicine, Massachusetts General Hospital, Boston, MA 02114, USA; Department of Biostatistics, University of Washington, Seattle, WA 98195, USA; Cardiovascular Health Research Unit, University of Washington, Seattle, WA 98195, USA; Cardiovascular Health Research Unit, University of Washington, Seattle, WA 98195, USA; Department of Medicine, University of Washington, Seattle, WA 98195, USA; Department of Epidemiology, University of Washington, Seattle, WA 98195, USA; Cardiovascular Health Research Unit, University of Washington, Seattle, WA 98195, USA; Department of Medicine, University of Washington, Seattle, WA 98195, USA; The Institute for Translational Genomics and Population Sciences, Department of Pediatrics, The Lundquist Institute for Biomedical Innovation at Harbor-UCLA Medical Center, Torrance, CA 90502, USA; Division of Public Health Sciences, Fred Hutchinson Cancer Center, Seattle, WA 98109, USA; Division of Public Health Sciences, Fred Hutchinson Cancer Center, Seattle, WA 98109, USA; Department of Epidemiology, Erasmus Medical Center, 3015 GD Rotterdam, The Netherlands; Nuffield Department of Population Health, University of Oxford, Oxford OX1 2JD, UK; Department of Epidemiology, Erasmus Medical Center, 3015 GD Rotterdam, The Netherlands; Department of Epidemiology, Erasmus Medical Center, 3015 GD Rotterdam, The Netherlands; Nuffield Department of Population Health, University of Oxford, Oxford OX1 2JD, UK; Programs in Metabolism and Medical & Population Genetics, Broad Institute of Harvard & Massachusetts Institute of Technology, Cambridge, MA 02142, USA; Division of Endocrinology, Diabetes and Metabolism, Department of Medicine, Cedars-Sinai Medical Center, Los Angeles, CA 90048, USA; Department of Medicine, Harvard Medical School, Boston, MA 02115, USA; Programs in Metabolism and Medical & Population Genetics, Broad Institute of Harvard & Massachusetts Institute of Technology, Cambridge, MA 02142, USA; Center for Genomic Medicine and Diabetes Unit, Massachusetts General Hospital, Boston, MA 02114, USA; Department of Medicine, Harvard Medical School, Boston, MA 02115, USA; Programs in Metabolism and Medical & Population Genetics, Broad Institute of Harvard & Massachusetts Institute of Technology, Cambridge, MA 02142, USA; Division of General Internal Medicine, Massachusetts General Hospital, Boston, MA 02114, USA; The Institute for Translational Genomics and Population Sciences, Department of Pediatrics, The Lundquist Institute for Biomedical Innovation at Harbor-UCLA Medical Center, Torrance, CA 90502, USA; Center for Public Health Genomics, Department of Public Health Sciences, University of Virginia, Charlottesville, VA 22903, USA; Department of Biostatistics, Boston University School of Public Health, Boston, MA 02215, USA; Department of Medicine, Harvard Medical School, Boston, MA 02115, USA; Center for Genomic Medicine and Diabetes Unit, Massachusetts General Hospital, Boston, MA 02114, USA; Division of General Internal Medicine, Massachusetts General Hospital, Boston, MA 02114, USA

**Keywords:** type 1 diabetes, type 2 diabetes, genetics, polygenic score

## Abstract

**Context:**

Both type 1 diabetes (T1D) and type 2 diabetes (T2D) have significant genetic contributions to risk and understanding their overlap can offer clinical insight.

**Objective:**

We examined whether a T1D polygenic score (PS) was associated with a diagnosis of T2D in the Cohorts for Heart and Aging Research in Genomic Epidemiology (CHARGE) consortium.

**Methods:**

We constructed a T1D PS using 79 known single nucleotide polymorphisms associated with T1D risk. We analyzed 13 792 T2D cases and 14 169 controls from CHARGE cohorts to determine the association between the T1D PS and T2D prevalence. We validated findings in an independent sample of 2256 T2D cases and 27 052 controls from the Mass General Brigham Biobank (MGB Biobank). As secondary analyses in 5228 T2D cases from CHARGE, we used multivariable regression models to assess the association of the T1D PS with clinical outcomes associated with T1D.

**Results:**

The T1D PS was not associated with T2D both in CHARGE (*P* = .15) and in the MGB Biobank (*P* = .87). The partitioned human leukocyte antigens only PS was associated with T2D in CHARGE (OR 1.02 per 1 SD increase in PS, 95% CI 1.01-1.03, *P* = .006) but not in the MGB Biobank. The T1D PS was weakly associated with insulin use (OR 1.007, 95% CI 1.001-1.012, *P* = .03) in CHARGE T2D cases but not with other outcomes.

**Conclusion:**

In large biobank samples, a common variant PS for T1D was not consistently associated with prevalent T2D. However, possible heterogeneity in T2D cannot be ruled out and future studies are needed do subphenotyping.

It has become increasingly clear that type 1 diabetes (T1D) and type 2 diabetes (T2D) cannot be accurately distinguished on the basis of clinical features alone [1]. This is particularly true for adolescents and young adults and it is thought that up to 15% of young adults with diabetes are wrongly classified and incorrectly treated [2]. Obesity is now widespread across all age groups [3] and can no longer be used to distinguish T1D and T2D, as people with T1D are equally susceptible to the same environmental and genetic risk factors that lead to obesity. Additionally, autoimmune diabetes can occur at any age, even at older ages [4, 5]. Accurate classification of the type of diabetes is essential, as management approaches and risk of comorbidities and complications differ between the 2 diseases [6‐8]. Failure to accurately diagnose T1D could lead to life-threatening diabetic ketoacidosis, and inaccurate diagnosis of T2D could lead to unnecessary treatment with insulin when other more appropriate options may be available.

However, while there is overlap in clinical features, it remains unclear if there is any overlap in the genetic predisposition for developing the two forms of diabetes. T1D has a significant heritable risk estimated to be between 40% and 60% based on familial and twin studies with approximately 50% of this heritability attributable to the HLA region and loci from other parts of the genome making smaller additional contributions to disease risk [9‐13]. Similarly, T2D has a polygenic inheritance pattern and a heritability estimate between 30% and 70% with hundreds of variants that have been reported to be associated with disease risk based on genome-wide association studies (GWASs) and exome sequencing studies, but with each individual variant only contributing a small increase in disease risk [14, 15]. In terms of genetic overlap, individual loci such as *INS*, *TH*, *CTRB1*, *CENPW*, *HLA-DRA* [15], *GLIS3* [16], *MTNR1B*, *HNF1A* [17], and *POU5F1-TCF19* [15] been reported to be associated with both T1D and T2D in large-scale GWAS. However, whether there is genetic overlap between T1D and T2D or pleiotropy at these loci remains largely unknown.

Identifying any genetic overlap between T1D and T2D has the potential to offer new biological and clinical insight through a better understanding of diabetes mellitus heterogeneity and can help predict clinical courses. Our main objective was to evaluate whether a genetic predisposition for developing T1D, modeled using a comprehensive T1D polygenic score (PS) composed of genetic variants within and outside the HLA region, was associated with prevalent T2D in older adults from the Cohorts for Heart and Aging Research in Genomic Epidemiology (CHARGE) consortium. We validated CHARGE association findings in the clinically based Mass General Brigham (MGB) Biobank, and in secondary analyses, tested associations of the T1D PS with T2D clinical characteristics.

## Materials and Methods

We examined a T1D PS in adults with a clinical diagnosis of T2D from the Cohorts for Heart and Aging Research in Genomic Epidemiology (CHARGE) consortium and validated our findings in the MGB Biobank.

### Study Populations

#### The cohorts for heart and aging research in genomic epidemiology (CHARGE) consortium

We included participants with T2D from the following CHARGE cohorts: Framingham Heart Study (FHS) [18‐20], Cardiovascular Health Study (CHS) [21], Multi-Ethnic Study of Atherosclerosis (MESA) [22], and the Women's Health Initiative (WHI) [23]. Analysis was restricted to participants of European ancestry. Cases were selected based on the presence of any of the following criteria: age of onset of diabetes ≥45 years when information was available to minimize contamination with T1D in younger adults, and at least 1 of the following based on data availability in the individual cohorts: fasting plasma glucose ≥7 mmol/L (126 mg/dL), random glucose or 2-hour glucose on oral glucose tolerance test >11.1 mmol/L (200 mg/dL), HbA1C ≥6.5%, or the use of an oral medication to treat diabetes. Data from 14 169 controls were used in the analysis, and controls had to satisfy the following criteria based on available phenotypes in the individual cohorts: no self-report or physician diagnosis of diabetes, fasting plasma glucose <5.6 mmol/L (100 mg/dL), 2-hour glucose on oral glucose tolerance test <7.8 mmol/L/dl (140 mg/dL), random glucose <11.1 mmol/L (200 mg/dL), HbA1C <5.7%, absence of pancreatic autoantibodies, and could not be on any medication used to treat diabetes. Institutional Review Board and appropriate oversight committees approved the study in each participating cohort and all participants provided written informed consent including consent for use of genetic information.

#### Mass General Brigham Biobank

The MGB Biobank (formerly Partners HealthCare Biobank) [24] maintains blood and DNA samples from more than 60 000 consented patients seen at MGB hospitals, including Massachusetts General Hospital, Brigham and Women's Hospital, McLean Hospital, and Spaulding Rehabilitation Hospital, all in the greater Boston area (USA). Details on the recruitment process have been previously described [24]. MGB Biobank participants provided written informed consent for the use of their samples and data in broad-based research. T2D status was defined based on “curated phenotypes” developed by the Biobank Portal team using both structured and unstructured electronic medical record data and clinical, computational, and statistical methods. Natural Language Processing was used to extract data from narrative text. Specifically, the MGB Biobank uses the eMERGE phenotyping algorithm [25], which is a machine learning–based algorithm using the PheCAP method [26], a high-throughput semisupervised phenotyping pipeline. Chart reviews by disease experts helped identify features and variables associated with particular phenotypes and were also used to validate results of the algorithms. The process produced robust phenotype algorithms that were evaluated using metrics such as sensitivity, the proportion of true positives correctly identified as such, and positive predictive value, the proportion of individuals classified correctly as cases out of all those classified as cases by the algorithm [27]. Cases were individuals determined by the “curated disease” algorithm employed above to have T2D with positive predictive value ≥99% and age ≥45 years. Controls were individuals determined by the “curated disease” algorithm employed above to have no history of T2D with negative predictive value of 99%.

### Clinical Measurements

#### CHARGE consortium

##### Exposure

The T1D PS is the exposure in all the analysis. Genotyping, imputation, and quality control were done independently by each cohort and are described elsewhere (Table S1 [28]). An imputation r [2] threshold of >0.80 was used for all the cohorts.

##### Outcomes

(1) The primary outcome was prevalent T2D vs non-T2D, defined using standard criteria by each cohort using available data (Table S1 [28]). (2) The association between the T1D PS and quantitative traits was evaluated as secondary analyses. The methods used to collect phenotypes in each cohort are described elsewhere (Table S1 [28]). Participants on medications to treat diabetes were excluded from the HOMA-β and HOMA-IR measurements. CHS did not participate in the quantitative trait analysis because the phenotypes were not consistently available at the time of diabetes onset.

#### MGB Biobank

We validated our results using the T1D PS in 2256 T2D cases and 27 052 controls from the MGB Biobank.

##### Exposure

The T1D PS was the primary exposure. Genomic data were generated with the Illumina Multi-Ethnic Genotyping Array and genotypes were phased with SHAPEIT2 [29]. Imputation was done with the Michigan Imputation server, using the Haplotype Reference Consortium [30] as the reference panel. We excluded variants with an imputation r [2] <0.5 and a minor allele frequency <0.005.

##### Outcomes

The primary outcome was T2D status as defined in the inclusion criteria. Quantitative trait analyses were not done in the MGB Biobank.

### Genotyping, Imputation and PS Construction

Details on genotyping, quality control, and imputation for the individual cohorts are provided elsewhere (Table S1 [28]). The PS was calculated as the sum of the number of T1D risk-raising alleles (0, 1, or 2) per distinct single nucleotide polymorphism (SNP), weighted by the effect size of each allele using weights from published literature [10]. The SNPs used in the T1D PS were robustly associated variants from GWASs [9] and included a total of 79 independent SNPs, 27 mapped to the HLA regions and 52 to the non-HLA regions. The complete list of SNPs is included elsewhere (Table S2 [28]). The PS was developed using data from 6481 cases and 9247 controls from the Type 1 Diabetes Genetics Consortium [receiver operating characteristic area under the curve (ROC AUC) 0.927 for T1D discrimination] and is well validated (ROC AUC 0.921 in the UK Biobank and 0.73 in the TEDDY study) [31, 32]. If a SNP was not directly genotyped in the individual cohort, an imputed SNP of good imputation quality (imputation r [2] >0.8) was used. If a directly genotyped or imputed SNPs are not available, the SNP was omitted, and the GRS was scaled appropriately.

### Statistical Analysis Including Type 1 Error Rate for the Study

For the analyses of the effect of the PS on clinical outcomes, a linear regression model or linear mixed effects model (in samples with related individuals) were used for continuous outcomes. A logistic regression model with robust standard error estimates using generalized estimating equations for related samples was used for the analyses of binary outcomes and for the T1D PS association analysis in cases and controls. Covariates included age and sex when applicable. Analyses were conducted separately in each cohort followed by meta-analyses of effect estimates using an inverse variance fixed effect approach after conducting a test for heterogeneity and confirming that it was not significant. For the primary outcome of association of T1D PS with T2D status, the *P* value threshold for statistical significance was set at .05. The quantitative trait analyses were considered as secondary exploratory analyses.

## Results

**Figure 1.**
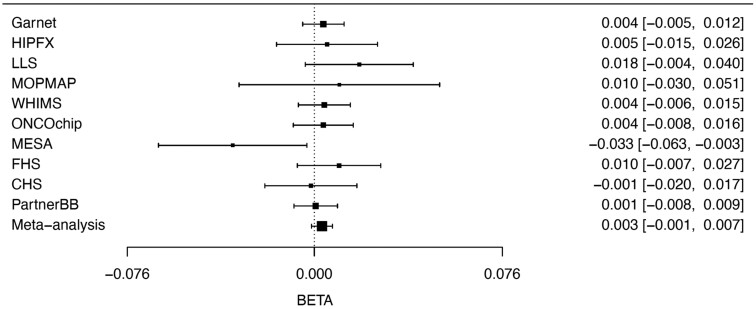
Forest plot showing association of the T1D PS with T2D in the individual cohorts and in the meta-analyses. Garnet, HIPFX, LLS, MOPMAP, WHIMS, and ONCOCHIP are Women's Health Initiative (WHI) substudies; MESA, Multi-Ethnic Study of Atherosclerosis; FHS, Framingham Heart Study; CHS, Cardiovascular Health Study; PartnerBB, Mass General Brigham Biobank.

A total of 13 792 T2D cases were included in the analysis of the association of the T1D PS with T2D status with 817 cases from FHS, 588 cases from CHS, 195 cases from MESA, and 12 162 cases from WHI. The mean age of the cases was 65.9 years (SD 9.9), 94% were female and the mean body mass index (BMI) was 30.1 (SD 5.9) kg/m^2^. The characteristics of the cases by individual cohort are shown in Table 1. A total of 14 169 controls from the various cohorts were included in the analyses. The T1D PS was not significantly associated with T2D case status (OR 1.003 per 1 SD increase in PS, 95% CI 0.999-1.006, *P* = .15). The forest plot showing association of the T1D PS with T2D in the individual cohorts and in the meta-analyses is shown in Fig. 1. In an exploratory analysis, we examined the associations of the HLA and non-HLA components of the PS separately with T2D and found an association with the partitioned HLA PS (HLA PS OR 1.02, 95% CI 1.01-1.03, *P* = .01; non-HLA PS OR 1.00, 95% CI 0.99-1.01, *P* = .45). We note that the association between the partitioned HLA PS and T2D was observed in a single cohort and did not replicate in MGB Biobank (Table S4 [28]).

**Table 1.** Characteristics of participants with T2D

Mean (SD)	FHS	CHS	MESA	WHI
n	817	588	195	12 192
Age, years	61.0 (9.5)	72.7 (5.4)	65.5 (9.7)	66.3 (6.6)
% Female	44.7	58.2	41.5	100
BMI, kg/m^2^	31.7 (6.2)	27.9 (4.6)	31.1 (5.7)	28.4 (5.8)
HbA1C, %	6.4 (1.0)	N/A	6.9 (1.3)	N/A
PS, total	95.7 (5.7)	99.6 (5.4)	97.0 (5.4)	95.6 (5.4)
PS, HLA	33.3 (2.7)	35.3 (2.4)	33.8 (2.6)	33.7(2.6)
PS, Non-HLA	62.1 (4.7)	61.9 (5.7)	61.3(3.2)	60.1 (4.2)

Abbreviations: BMI, body mass index; CHS, Cardiovascular Health Study; FHS, Framingham Heart Study; MESA, Multi-Ethnic Study of Atherosclerosis; N/A, not available; PS, polygenic score; T2D, type 2 diabetes; WHI, Women’s Health Initiative.

To further validate our findings, we also examined the association of the T1D PS with T2D status in 2256 T2D cases ≥45 years and 27 052 controls from the MGB Biobank. Mean age of the cases was 6.3 years (SD 16.7), 52.4% were female, mean BMI was 28.3 (SD 6.5) kg/m^2^, and mean HbA1C % was 5.9 (SD 1.1). After accounting for age and sex, the T1D PS was not associated with T2D in MGB Biobank (OR 0.999, 95% CI 0.991-1.006, *P* = .87).

In secondary analyses, we also evaluated the association of the T1D PS with metabolic-related quantitative traits in 5228 T2D cases in whom the data were available (Table 2). The T1D PS was significantly associated with the use of insulin (OR 1.007, 95% CI 1.001-1.012, *P* = .03) and was not significantly associated with other clinical characteristics. The T1D PS was not associated with T2D age of onset, BMI, HbA1C, HOMA-β, or HOMA-IR after excluding participants on medications for T2D.

**Table 2.** Association of the T1D PS with clinical outcomes of interest

	Sample size	Parameter estimate	Standard error	*P* value
Age of onset of diabetes	5312	0.005	0.015	.74
ln BMI	5289	−0.001	0.0004	.16
HbA1C	774	−0.003	0.008	.65
ln HOMA-IR	781	0.003	0.005	.49
ln HOMA-B	780	0.007	0.005	.15
Insulin use	4479	0.007	0.003	.03
Any medication use	4479	0.001	0.004	.73

Abbreviations: BMI, body mass index; HOMA, Homeostasis Model Assessment.

## Discussion

We evaluated a comprehensive 79 SNP T1D PS representing both the HLA and non-HLA regions and showed that there was no association between the T1D PS and T2D status in more 13 000 cases in the CHARGE consortium and over 2000 cases in the MGB Biobank. We then examined the contribution of the partitioned HLA and non-HLA PS and found an association between the partitioned HLA PS and T2D in CHARGE that did not replicate in MGB Biobank. Upon examining the effect estimates of individual cohorts, we noted that the association was only significant in a single cohort. Inclusion of T2D cases in the T2D group (contamination) could have affected results in this cohort, but we cannot exclude the possibility of genetic overlap between T1D and an autoimmune subtype of diabetes among T2D cases. Pancreatic autoantibodies, which we lacked in our cohorts, would have allowed us to clarify whether the association was driven by latent autoimmune diabetes of adults (LADA). We also recognize that the CHARGE sample was larger and thus, had more power to detect subtle associations. Future investigations with larger replication samples are needed to clarify the genetic overlap, if any, between T1D and subtypes of T2D. In secondary analyses, we showed that the total T1D PS was associated with insulin use in participants with T2D but not with other quantitative traits that have been considered characteristic of T1D.

A potential biological overlap between T1D and T2D had been hypothesized because impairment in insulin secretion is a key factor in the pathogenesis of both diseases. However, despite common characteristics in the clinical presentation, the biological mechanisms underlying T1D and T2D pathogenesis remain distinct. Previous studies have found associations between T1D and known T2D loci including in the melatonin receptor 1B *(MTNR1B)* and *HNF1A* [17], and results from a multiancestry GWAS for T2D showed that *POU5F1-TCF19* and *HLA-DRA* within the major histocompatibility complex was associated with T2D in addition to *INS*, *TH*, *CTRB1*, and *CENPW* [15] (Table S3 [28]). Similarly, *GLIS3* was identified as a susceptibility risk locus for both T1D and T2D in GWAS and decreased expression of *GLIS3* may contribute to both forms of diabetes by favoring β-cell apoptosis [16]. These loci appear to impact T1D and T2D risk through different mechanisms and do not indicate genetic overlap between T1D and T2D. A condition with possible genetic overlap is LADA. As patients with LADA present in adulthood and can have slower deterioration in their glycemia than T1D, they can be misdiagnosed with T2D early in their disease course and treated with oral agents before developing insulin dependency over time. A GWAS of 2634 adults with LADA and 5497 controls showed that while genetic signals were principally shared with T1D with attenuated potency of key HLA haplotypes, there were positive genetic correlations with both T1D and T2D [33]. Whether this represents true genetic overlap between distinct pathophysiological entities or contamination of T1D or T2D cases in the LADA dataset remains unclear. However, it is possible that pleiotropic variants that are associated with both T1D and T2D contribute to unique diabetes genetic subsets as there could be multiple pathways toward dysglycemia in T2D that should be explored in future work. However, our study shows that there is not a consistent effect across all genetic loci.

T2D PSs have not been shown to significantly improve clinical models for disease prediction [34, 35]. However, a T1D PS has a better predictive ability for T1D due to the strong effect sizes of the SNPs tagging the HLA region [36]. In a study by Oram et al, a 30-SNP T1D PS was evaluated in young adults with T1D and T2D diagnosed between 20 and 40 years of age in the Wellcome Trust Case-Control Consortium. Their results showed that a high T1D PS was indicative of T1D and a low T1D PS was indicative of T2D, with significant overlap between the distributions [36]. Our study has differences from Oram et al study, particularly in the method of case ascertainment. People in the WTCCC cohort were selected if T2D was diagnosed at an age greater than 25 years but less than 75 years. In CHARGE, we used a more stringent inclusion criteria of age greater than 45 years to reduce contamination of T2D cases with T1D. A study in the UK Biobank showed that 40% of autoimmune diabetes can occur after the age of 30 years [37]. Oram et al also used the absence of the GAD -65 antibody as an inclusion criterion. However, GAD -65 positivity is present in only 70% to 80% of T1D cases and participants could have been positive for the other autoantibodies, including insulinoma antigen 2 (IA2), islet cell 512, and Zinc transporter -8 antibodies [37, 38]. Overall, it appears that late-onset T1D or LADA can masquerade as T2D in adulthood but strong evidence for association of T1D genetic risk with T2D is lacking.

In secondary analyses, our results showed that the T1D PS was associated with insulin use in participants with T2D but not with other clinical characteristics. This may represent contamination of true T1D or LADA cases among the T2D cases ascertained in our study. It is also possible that individuals with a higher genetic burden for T1D have a greater degree of insulin deficiency, resulting in the need for treatment with insulin earlier in the disease. However, this result should be interpreted with caution as the remaining analyses did not show significant associations.

A major strength of this study is the use of a comprehensive 79 SNP score that improves upon earlier T1D PSs with fewer SNPs [36]. This is particularly pertinent for evaluation of the risk conferred in the HLA region because of the complexity of the region. The impact of the HLA region on T1D risk has been localized to HLA-DRB1 position 57, HLA-DRB1 position 13, and HLA-DRB1 position 71. Together, these 3 positions explain 90% of the risk in the DRB1-DQA1-DQB1 locus [12]. We recognize that, while non-HLA regions have some measurable association with T1D in large-scale GWAS, their contribution to T1D risk is smaller than the HLA region; still, our study confirms that common variant polygenic risk for T1D that considers both HLA and non-HLA regions do not contribute to T2D risk. An additional strength of the study is our independent validation of the results in the MGB Biobank. Our study also had limitations. Analyses were limited to participants of European ancestry. This was done as discovery of the T1D SNPs was performed in populations of European ancestry and therefore the PS may not accurately measure T1D risk in other populations. Contamination of T2D cases with T1D cases was possible as we did not have pancreatic autoantibodies in all cohorts. While this may explain the association of the T1D PS with insulin use in a single cohort, contamination in our other analyses would have biased our results away from the null. Given that we report null results, our interpretation of the results remains unchanged. Additionally, the lack of association between the T1D PS and T2D was confirmed in the MGB Biobank, a patient cohort that was likely more susceptible to contamination. Another demographic detail to note is that 94% of our cohort was female as the majority of cases came from the WHI cohort. However, we verified our results in the MGB Biobank which has a more balanced distribution of sex. Another potential consideration is the performance of our PS compared with recent scores that had considered multiplicative interaction effects in the major histocompatibility complex [35]. In the FHS cohort with 817 participants, we found that <2% of participants carried the high-risk DR-DQ haplotype combinations. Therefore, we would unlikely detect a strong association of the T1D PS with T2D even when accounting for these interactions. To determine whether our study was able to detect a modest association between the T1D PS and T2D, we performed a power calculation and showed that, in a study with a minimum of 13 791 T2D cases and an equivalent number of controls, using 2-sided α = .05, we had at least 80% power to detect an odds ratio of 1.034 per 1 SD increase in T1D PS. Therefore, if an association were to be detected with larger sample sizes, the effect on T2D would likely be extremely small and of questionable clinical relevance. However, it is still possible that certain subtypes of T2D do have genetic overlap with T1D but were underrepresented in our study.

In summary, in large population cohorts and biobank samples, common variant genetic risk for T1D was not consistently associated with prevalent T2D. Future studies with detailed clinical measures and in particular pancreatic auto-antibody status, are needed to further investigate the heterogeneity and possible sub-phenotypes of T2D.

## Data Availability

Some or all datasets generated during and/or analyzed during the current study are not publicly available but are available from the corresponding author on reasonable request.

## Current Affiliation

Josée Dupuis, Department of Epidemiology, Biostatistics and Occupational Health, McGill University, Montreal, Quebec, Canada.

## Abbreviations

BMI: body mass index
CHS: Cardiovascular Health Study
FHS: Framingham Heart Study
GWAS: genome-wide association study
HOMA: Homeostasis Model Assessment
LADA: latent autoimmune diabetes
MESA: Multi-Ethnic Study of Atherosclerosis
PS: polygenic score
SNP: single nucleotide polymorphism
T1D: type 1 diabetes
T2D: type 2 diabetes
WHI: Women's Health Initiative
